# Combining Outreach and Cross-sectional Research to Gather Children’s Soil Values in Aotearoa New Zealand: Protocol for the Mixed Methods Soilsafe Kids Program

**DOI:** 10.2196/43390

**Published:** 2023-03-29

**Authors:** Sophia Tsang, Victoria Egli, Bridget Crawshaw-Mclean, Lianne Edwards-Maas, Melanie Kah, Emma Sharp

**Affiliations:** 1 Te Pū Ao | GNS Science Auckland New Zealand; 2 Te Kura Mātai Taiao | School of Environment Waipapa Taumata Rau | University of Auckland Auckland New Zealand; 3 Te Kura Naahi | School of Nursing Waipapa Taumata Rau | University of Auckland Auckland New Zealand; 4 Te Pūnaha Matatini Centre of Research Excellence hosted by the University of Auckland Auckland New Zealand

**Keywords:** soil values, environmental education, child-centric research, child-centered teaching and learning, transdisciplinary, Tāmaki Makaurau Auckland, soil, school, student, child, environment, nature, natural

## Abstract

**Background:**

Soil underpins most terrestrial systems; hence, its degradation should concern everyone. In 2021, Soilsafe Aotearoa surveyed the adult population of New Zealand about how they value soil, particularly values related to how they care about and are concerned about soil. Pursuant to this study, Soilsafe Kids (the outreach branch of Soilsafe Aotearoa) developed a combined research and outreach program to collect a supplemental data set of children’s soil values, so both adults’ and children’s voices can be considered when understanding the implications of different practices and how to care for presently “uncared for” or neglected soils in the future.

**Objective:**

The program not only asks primary school students about their soil values but also aims to teach them about soil from many disciplinary perspectives to enhance their understanding and awareness of soil, and, more broadly, for knowledge production.

**Methods:**

Here we describe the research protocol used in this Soilsafe Kids program. This program uses surveys (in the form of worksheets), focus groups (introduced as group discussions), and art projects to learn what children think about soil in Tāmaki Makaurau Auckland. We have received ethics approval from the University of Auckland’s Human Participants Ethics Committee (reference number 23556) on March 25, 2022, for 3 years.

**Results:**

We have begun recruiting participants and delivering the Soilsafe Kids program in schools across Tāmaki Makaurau Auckland. Our data collection is ongoing with final student engagement in the first quarter of 2023. We expect to analyze data at the start of 2023 and to disseminate results later this year.

**Conclusions:**

Once this study is complete, we will disseminate the final results to the research community, stakeholders, and the local community through conference presentations, journal articles, hui (meetings), on our website, and in art exhibits. We note that although Tāmaki Makaurau Auckland is home to the majority of people living in Aotearoa New Zealand, the Auckland region only represents a small portion of Aotearoa New Zealand’s land, and findings are not generalizable to Aotearoa New Zealand as a whole.

**International Registered Report Identifier (IRRID):**

DERR1-10.2196/43390

## Introduction

While encounters with soil tend to be everyday and be mundane, many of us do not think deeply, or much at all, about its connections or its diverse values (eg, as calculative, emotive, or utilitarian). Soil underlies spaces we interact with and is a medium in which animate entities grow. Additionally, soil is a site of biodiversity and recreation and has its own intrinsic value. Particularly important for the context of this study are Māori Indigenous values of soil and the broader environment, with vital connection to the *whenua* (land) and *atua* (creators) [1], and the context of largely conventional “valuation” of soil taking place by way of settler-colonial knowledge frameworks, with soil being both *tīpuna* (ancestor) and stolen land simultaneously. Globally, soil quality is decreasing for reasons such as its use as a platform for development, its overuse in food and resource productivity, and as a deliberate or accidental sink of waste, holding contaminants such as metals and pesticides. Nonetheless, soil is a foundation to human settlements, we relate to it in diverse ways, and food is generated out of soil. In fact, some of us produce that food at home. Domestic and community gardening has long been a solution for populations in Tāmaki Makaurau (Auckland), and Aotearoa New Zealand (hereinafter “NZ”), to gain access to affordable, adequate, and culturally appropriate food supply [2]. Of concern, then, is the significant scholarship that discuss soil degradation as the number 1 threat to food security and environmental sustainability; for example, Krzywoszynska [3] and Osman [4,5].

In particular, there is concern for soil degradation in the form of soil metal contamination. Lead is one metal of notoriety in urban soils because of its toxicity, persistence, and ubiquity in cities [6,7]. In urban locations, there is a legacy of leaded petrol use in vehicles, industrial pollution, waste incineration, and leaded paint usage [8-11]. While the use of paints containing lead was outlawed in New Zealand between 1965 and the early 1980s (different paints were banned at different times), older homes may still therefore contain lead-based paint [12], and house paint that is poorly maintained will flake into surrounding soil. Contaminated garden soils are a global problem [13], as demonstrated in Canada [14], Denmark [15], France [16], Hungary [11], Italy [16,17], and the United States [18,19]. Other heavy metals—such as mercury, arsenic, chromium, and cadmium—have also been found in soils, including soil that is used for growing food, thus creating an exposure pathway for metal absorption by humans [20]. Soil contamination can also be a multigenerational problem, where the contamination does not “go away” but rather is retained in situ within soils until disturbed or mobilized (eg, through water, by wind, manually by humans, or where it is taken up by plants). There are numerous links to the importance of children learning about soil quality, including potential sources of contamination and an appreciation of less documented benefits that soil brings that relate to its intrinsic (nonutilitarian) and well-being values [21].

Soilsafe Kids is a research and outreach project that sits under the umbrella of Soilsafe Aotearoa [22]. Soilsafe Aotearoa launched in 2021 in NZ as a program to examine diverse soil values, including those that are scientific, cultural, ecological, economic, political, and artistic. After a year of learning about why the public values soil and their concerns about soil, Soilsafe Aotearoa launched a citizen science project to test domestic garden soils for metal contaminants, free of charge, in response to the concerns raised [22], including a study on gardening values (eg, EL Sharp, F Porter, N Strawbridge et al, unpublished data, January 2023) and matters of soil care and concern. All respondents in this study of soil care and concern were adults.

This leads us to question “Who are the *public*?” Nearly a quarter of NZ’s population is younger than 15 years [23,24]. Although children spend more time outdoors than adults [25], we know very little about what children think, feel, or know about soil. In order to understand what the *broader* public values about soil, it is paramount that we understand what children value, not just adults; further, the diversity in the known and unknown perspectives of adults and children, rather than just an adult-child binary, is of interest here.

Soilsafe Kids launched in 2022, with keystone activities including a research study gathering “soil values” parallel to that undertaken by Soilsafe Aotearoa with adults, instead of children, and the opportunity for the families of the participating children to take part in Soilsafe Aotearoa’s heavy metals soil testing, along with an in-class educational component to teach children about factors that might lead to the risk of soil contamination or human exposure to contamination. Ultimately, this information would guide *whānau* (families) in their decision-making about whether their gardens are safe places to grow food, and, if not, what to do about it to reduce contaminant exposure, including government guidance regarding practices such as eliminating sources of contamination depending on the metal, growing food in commercially available soils that are brought to the site (not growing in ground soil), moving the location of the food garden, or reducing food or hand-to-mouth exposure to contaminants.

The delivery of a combined educational and research program offers diverse benefits to children, their communities, and researchers. First, it establishes a reciprocal relationship with participants, where we provide knowledge and demonstrations of practice as well as obtaining data from participants [26], demonstrating the value of the knowledge imparted to us. Second, it prioritizes and highlights the value of knowledge dissemination in ethical research practice by emphasizing children’s right to be heard and seriously considering their views in relation to policy and planning [27]. Third, educational and research activities offered jointly mean that knowledge can be coproduced in the community and knowledge previously produced can be returned to communities who can use it. The aforementioned benefits of combining teaching and research into 1 program, as the Soilsafe Kids program does, are important given children’s increasing roles and participation in contemporary issues, as evidenced by the COVID-19 pandemic [28] and the climate crisis [29].

This Soilsafe Kids program is intended to run over 2-3 days depending on the context of the individual school’s needs. Our research question is, “what do children (aged 6-10 years) think about soil, and how do they value it?”

The program is composed of 8 workshops designed and run by social, physical, and health scientists with support from scientists from the New Zealand Crown Research Institutes GNS Science and AgResearch, charities Oke and Garden to Table Trust, 2 community artists, a Māori tikanga advisor, and a Royal Society of New Zealand Te Apārangi Teaching Fellow. The benefits of this type of transdisciplinary research—that encourages children to raise awareness in their communities through environmental activism—include unique educational opportunities [28] and embracing education as a driver for sustainable development [30].

To address our research question, the program is planned to include surveys at the beginning and end of the program, supplemented with focus groups and drawings throughout the workshops. Here, we describe our study design and outline how we will analyze our data and disseminate our findings, allowing us to consider the potential impact of this work.

## Methods

Soilsafe Kids aims to understand what children know about soil and how they value it. This takes a child-centered approach [31,32] throughout our workshop series with 8 primary school classes in Tāmaki Makaurau Auckland. Class sizes are planned for approximately 30 students, resulting in a convenience sample of ~240 participants. Data collection and student engagement began in 2022 and is intended to last approximately 9 months, with dissemination of results intended for early 2023.

### Ethical Considerations

The University of Auckland’s Human Participants Ethics Committee approved this study (reference number 23556) on March 25, 2022, for 3 years. Principals, teachers, and families will provide informed consent via written consent forms; all children (ie, “the participants”) will provide assent by signing forms as their reading level precludes truly informed consent. As required by ethics approval processes, Soilsafe Kids has developed participant information sheets (PIS), consent forms (CF), assent forms, and recruitment posters with specific wording and sections of information [33]. When writing the student versions, we were cognizant that we would be working with students who may have rudimentary reading skills, so these documents have larger text, less text, and a limited vocabulary to ensure that the information is child-friendly. During our first interactions with principals, we have received a few comments about the amount of reading that will be required of families. While the PIS and CF are a page each (front and back), they are dense documents. Even though these forms are a standard part of the ethics approval process, this has led to discussions about how to make the recruitment stage more approachable in the future. In each of these forms, we acknowledge that we will maintain the confidentiality of all participants in research outputs but that we cannot guarantee anonymity as participation will occur in a public manner (ie, in a classroom setting).

Our program was designed before young students were eligible to be vaccinated against COVID-19. To help ensure that we minimize the risk of introducing COVID-19 into classes, our in-school team is fully vaccinated and boosted against the virus. Although NZ’s vaccine mandate for teachers and staff has since dropped, we are maintaining a vaccination requirement for our in-school team to enter schools. We also respect that schools may not want visitors during periods of high COVID-19 transmission. Thus, we have the ability to transition our program fully outdoors or remotely—this would allow for students to learn about soil in an environment that prioritizes their health.

Although we strongly prefer that students participate in Soilsafe Kids’ research component, we understand that some families and students may not want to. Because Soilsafe Kids has both research and educational outreach objectives, we will ensure that students can partake in the educational component without participating in the research component. We have designed each workshop to accommodate this preference. For example, multiple focus groups will be held simultaneously with some groups being recorded as data while others being carried out as unrecorded discussions. Through simultaneous focus groups with the same topics and structure, looking around the classroom will not indicate who is and is not participating in the research. Such choices will help make our lessons more accessible to all students, regardless of their and their families’ feelings about participating in research.

All participants (including students, families, teachers, and principals) will have several opportunities to learn about our results. We will send a summary of school-specific results of the children’s values of care and concern for soil back to the principal within a few weeks of the final workshop. At the end of the year, we will host a webinar to describe our combined findings about children’s collective soil values. This format will enable our results to be disseminated, irrespective of people’s locations or the COVID-19 restrictions at the time, thus allowing us to highlight students’ communication outputs. Additionally, all participants will have the opportunity to request the study results in written form. By offering multiple ways of summarizing the study results, we hope to be able to reach a wider audience that is able to engage in a way that is appropriate to them. Results of the citizen science metal testing of home garden soil, as per the Soilsafe Aotearoa’s own protocol, are only shared back to the citizen science participant and not the wider public. While this protocol is counter to many citizen science movements that are working toward “open” science, data, and access, the choice to manage data in this way was made because of the risks of this openness [34]—in this study, the politics and potential harmful effects of particular locations being labeled as “contaminated places.” Despite the plans to communicate this, there is awareness that some may choose not to participate, given the unequal impacts of this type of environmental monitoring, as well as the known reduced participation of more marginalized communities in community science due to limitations of time and resources and possible surveillance and engagement fatigue concerns among Indigenous communities [34].

All participating schools were given gift cards worth NZ $500 (US $316.89 [exchange rate as on February 14, 2023, is indicated here]) of gift cards, while each student in the participating class (regardless of research participation status) received a NZ $30 (US $18.83) gift card to Booksellers Aotearoa New Zealand. By providing every student with a gift card, we ensure that we do not disclose the participation (or nonparticipation) status of a student.

### Participant Recruitment

Participant recruitment is planned in 3 stages at each school. Initially, using a combination of our networks and cold approaches to schools, principals are approached with a recruitment flier, a PIS, and a study protocol document [33]. This targeted recruitment approach will ensure that our sample is composed of a range of geographical locations and settings, communities of low and high socioeconomic status, and an ethnic distribution that reflects Tāmaki Makaurau Auckland. At this point, we are happy to address any questions either electronically or in person as in keeping with the NZ Ministry of Health’s COVID-19 public health measures. Once the principal or school’s board of trustees has decided that their school will participate and signed a CF, we will share a recruitment flier, PIS, and the study protocol with the principal and request that their front office share these documents with appropriate teachers. These second-stage documents will provide teachers general information about Soilsafe Kids and address how students can be part of the teaching and research components of the program, just the teaching components, or abstain altogether. With a signed teacher CF, the third stage of recruitment will commence and focus on families and students. Teachers will be provided with recruitment fliers, PIS, and consent and assent forms to distribute to parents and potential student participants.

As the potential student participants will be early readers, we have developed documentation equivalent to the adult documentation to build students’ confidence and responsibility. To ensure that learner-appropriate vocabulary has been used, a registered and student teacher both assessed all documents [35,36]. Students are participants if their principal, teacher, and caregiver all return signed CFs and have signed an assent form.

### Accessibility

Traditionally, schools in close proximity to research centers have more opportunities to work with researchers [37,38]. While this is convenient, it also means that students attending schools farther away have fewer interactions with researchers and are less likely to imagine themselves as researchers [39]. With this in mind, we will begin recruitment by reaching out to principals of suburban and rural schools first. We hope that this will provide different students an opportunity to participate in research and humanize researchers.

### Data Collection

This program is composed of 8 workshops that will be led by a team of researchers and expert practitioners. Each workshop is designed and will be led by a social, natural, or health scientist, an artist, or local charity. This diversity of backgrounds ensures that students gain a transdisciplinary understanding of soil. When possible (see the *Discussion* section for more information), the person who designed the workshop will also deliver it with support from other researchers.

The topics addressed on the first day of workshops include the following: an introduction, te ao Māori (the Māori worldview) on soil, composting, and how to sample soil for heavy metal analysis, as well as information on reducing exposure or introduction of contaminants into backyard soil. The topics addressed on the second day of workshops include the following: soil as an artistic medium, soil characteristics from a Western soil science perspective, and the relationship between food and soil. The second day ends with a science communication workshop in which students then create an output sharing the importance of soil and how to keep it healthy. Depending on the communication method, the Soilsafe Kids team will return for a third day to help the students share their soil messaging with their communities.

During the workshops, data are collected in the form of surveys presented as worksheets, art projects, focus groups that are labeled as “small group discussions,” a hands-up survey, and an interview with the teacher. As the students participating in Soilsafe Kids are still in early primary school (aged between 6 and 10 years), they may not be proficient readers or writers, so many of our activities revolve around art tasks. The Soilsafe Kids team will also write a critical researcher reflection, using the framework developed by Fook and Gardener [40] at the end of each day.

## Results

One of the goals of this program is to center students in knowledge production, so they realize their agency in the world. Thus, the final format of the data will be decided by the students. We anticipate using NVivo [41] to conduct automated and manual thematic analysis [42] to analyze the qualitative data [43], which will supplement our previous data set of adults’ soil values.

### Public Involvement

Local practitioners and stakeholders are central to the formation and operation of Soilsafe Kids. We have partnered with local practitioners, and stakeholders from Soilsafe Kids’ inception and will continue to involve them in the conduct of workshops, depend on them as advisors in the data analysis phase, and provide inputs regarding the dissemination of the results and knowledge transfer stages of the project.

When we were initially designing this program, our artists, local charities, and Royal Society Te Apārangi Teaching Fellow helped decide and shape our workshop series. The Teaching Fellow’s guidance and 2 of the authors’ teacher education training help ensure that Soilsafe Kids is aligned with the local curriculum. These partnerships have continued to evolve and grow as we have developed the Soilsafe Kids program.

To help foster relationships with the schools, we intend on offering meetings with all principals, boards of trustees, and teachers who might want to run the Soilsafe Kids program. Our aspiration is that the people we meet with will become ambassadors for the program during family and student recruitment. We will request that teachers make the first approach to families to minimize the transfer of personal information and ensure that a known contact makes first contact with families.

This program was designed for students who are familiar with school formats, who may not yet see themselves as having the authority to teach others in their community. Our workshops will aim to shift this view, especially in the final science communication workshop. This is important because these students not only are providing their current views on soil but also will inherit values of caring for the environment and soil. Actions taken now will affect the state of the soil that they have to work with in the future, so children need to view the soil as something they can influence now, not just later on.

### Dissemination

Our anticipated dissemination can be categorized as targeting the research community, stakeholders (eg, Auckland Council, the Ministry of Education, schools, students and their families, environmental education groups, Auckland Regional Public Health, Auckland District Health Board, and environmental artists), and the local community. Our results will be disseminated to the research community at academic conferences and through peer-reviewed academic journal publications. We maintain relationships with stakeholders and offer periodic updates in meetings and by sharing our research results. To transfer our new knowledge to communities, we will conduct a webinar for participants and partners and post updates on our blog. Our lessons also aim to empower students to share their learnings with their communities about soil, which is why our last workshop focuses on science communication. Additionally, Soilsafe Aotearoa held several soil-themed art exhibitions in 2021. We would like to use students’ artistic outputs to host such galleries for the public in the future. Finally, we will invite students to participate in Soilsafe Aotearoa’s soil testing for heavy metals. The results from this testing will be disseminated back directly to students’ families who contributed soil samples with a special child–friendly insert explaining their results in child-friendly terms. The results will only be shared back to the citizen science participant who takes part in this project and not the wider public, as explained in the *Ethical Considerations* section above.

### Data Storage

Paper consent forms will be stored in a locked cabinet in the locked office assigned to one of the Soilsafe Kids coleads (Sophia Tsang, Victoria Egli, or Emma Sharp). All data for this project will be stored digitally to ensure that the entire research team has access and that data are regularly saved and backed up. Given that we have many Indigenous participants, the guidelines set out by Te Mana Raraunga | the Māori Data Sovereignty Network [44] have shaped our data storage processes. This includes only using document transfer and backup systems that have domestic servers. To ensure that none of our data are transferred offshore, we will use the University of Auckland’s Webdropoff Service and local (ie, internal to the University of Auckland) storage. More information about our data storage can be found in our data management plan [45].

## Discussion

### Expected Findings

The Soilsafe Kids program is unusual due to its transdisciplinary nature while also combining educational outreach and research aims. This protocol describes our considerations as we designed the program to teach early to middle primary school students in NZ about soil. This will enable us to use mixed methods to gather student soil values that can supplement ongoing Soilsafe Aotearoa work that investigates adult soil values.

This program was designed to be accessible to schools in rural areas and to students with low reading levels during the COVID-19 pandemic (2020 to present). While accessibility and the pandemic guided the development of this program, we also were focused on ensuring that we provide students with an age-appropriate experience that supports their developing leadership potential, which all students could participate in the educational program without the research component without having their nonparticipation status disclosed to classmates, and that we followed best practices as laid out by Te Mana Raraunga | the Māori Data Sovereignty Network [44]. In line with the transdisciplinary Soilsafe Kids team, we will collect both quantitative and qualitative data to be analyzed, and we aim to share our results widely among academic circles, policy makers, and the public. To do so, we will disseminate our work in a variety of manners.

### Limitations

Everyone’s opinions and values are influenced by our contexts irrespective of whether they are spatial, temporal, cultural, or many others. We recognize that the results of Soilsafe Kids are not generalizable for 2 primary reasons related to the students we are working with. Although Tāmaki Makaurau Auckland is the largest population center in NZ, it is very urban compared to the rest of the country. While we aim to include more rural schools than in most programs, our results will not be representative of the country as a whole due to the schools’ proximity to Tāmaki Makaurau Auckland. Additionally, students who are currently in early to middle primary school will be shaped by current events and contemporary culture; a student who is currently 8 years old likely views the world and soil differently than a 15-year-old. Thus, additional work must be designed and undertaken to increase the robustness of our data set to students of different backgrounds including settings and ages.

### Conclusions

The Soilsafe Kids Three-Day program is a comprehensive way to ask children to consider the soil under their feet thinking through multiple worldviews. Through this program, we will gather students’ soil values while also encouraging them to participate in Soilsafe Aotearoa’s heavy metals soil testing. This not only will provide students a better understanding of the health of their soil at home but also ensures that they know if their home gardens are safe places to be growing food. By testing each students’ soil individually and communicating their results back with considerations and suggestions for improvement where necessary, students and families can make informed decisions about their gardens and if the plants they would like to grow could pose a health concern due to heavy metal uptake.

## Abbreviations

CF: consent form
NZ: Aotearoa New Zealand
PIS: participant information sheet

## Data Availability

An extended protocol document can be found in Tsang et al’s study [33].
